# Influence of Graphene Oxide on Cement Hydration Products, Microstructure, and Mechanical Performance

**DOI:** 10.3390/ma19102037

**Published:** 2026-05-13

**Authors:** Lounis Djenaoucine, Álvaro Picazo, Miguel Angel de la Rubia, Jaime C. Gálvez, Amparo Moragues

**Affiliations:** 1 Department of Civil Engineering: Construction, School of Civil Engineering, Technical University of Madrid, C/Profesor Aranguren s/n, 28040 Madrid, Spain; miguelangel.rubia@upm.es (M.A.d.l.R.); jaime.galvez@upm.es (J.C.G.); amparo.moragues@upm.es (A.M.); 2 Department of Building Technology, Superior Technical School of Building, Technical University of Madrid, Avda. Juan de Herrera, 6, 28040 Madrid, Spain; a.picazo@upm.es

**Keywords:** graphene oxide (GO), cement paste, hydration process, calcium silicate hydrate (C–S–H), calcium hydroxide (CH), microstructure, mechanical properties, nanomaterials, durability enhancement

## Abstract

This study examines the effects of graphene oxide (GO) on the hydration behaviour, microstructure, and mechanical properties of Portland cement-based materials. Cement pastes and mortars incorporating GO at dosages of 0.0005%, 0.005%, and 0.05% by weight of cement were analysed through thermogravimetric analysis (TGA), X-ray diffraction (XRD), scanning electron microscopy coupled with energy-dispersive X-ray spectroscopy (SEM/EDS), and mechanical strength testing. TGA results indicate that GO exerts a time-dependent influence on cement hydration. At early ages, GO slightly retards hydration, evidenced by lower C–S–H and CH content in GO-containing samples at 2 and 7 days, attributed to water adsorption by its oxygen-containing functional groups. At later curing ages (28–90 days), TGA results show greater C–S–H and CH weight losses in GO-modified samples compared to the reference, consistent with GO acting as a water reservoir and nucleation site. XRD and SEM results confirm that GO incorporation leads to a reduction in CH crystal size, a denser and more homogeneous microstructure, and fewer pores and microcracks. Mechanical tests revealed that GO contents of 0.0005% and 0.05% produced the most significant improvements, with increases of up to 9% in compressive strength and 16% in flexural strength at 90 days compared with the control specimens. In summary, the incorporation of low GO dosages effectively refines cement microstructure, enhances long-term hydration, and improves mechanical performance, demonstrating GO’s potential as a strength- and durability-enhancing nanomaterial for cementitious composites.

## 1. Introduction

Nanomaterials have revolutionised many fields of science, construction, and civil engineering, due to their unique properties, such as small size, high reactivity, and high specific surface area. Indeed, the incorporation of nanomaterials into cement-based materials has demonstrated measurable improvements in mechanical and durability performance: nano-SiO_2_ additions have been reported to increase compressive strength, while carbon nanotubes have reduced chloride permeability, yielding unique advantages in mechanical properties [1,2,3] and durability [4,5]. These findings have motivated growing interest in two-dimensional nanomaterials, such as graphene oxide (GO), as reinforcing agents for cementitious composites.

Nanomaterials that are frequently incorporated into cement materials recently can be classified according to their dimensions into three types: zero-dimensional nanomaterials, such as nano-silica (SiO_2_) and nano-titanium dioxide (TiO_2_), whose effectiveness in enhancing the performance of cementitious materials has been demonstrated by numerous studies. However, due to their low aspect ratio, these nanomaterials cannot withstand nanoscale micro-cracks [6,7,8]. One-dimensional nanomaterials, such as carbon nanotubes (CNTs), are commonly employed due to their high reactivity at the nanoscale; even so, their tendency to aggregate in fresh concrete diminishes their influence [9]. Two-dimensional nanomaterials, such as graphene oxide (GO), are a novel class of this type. Numerous studies [10,11,12] suggest that, owing to its extraordinary mechanical properties and large aspect ratios, it may be the most trustworthy option for use as an improvement agent of cementitious composites.

Graphene oxide (GO) is a sheet-like graphene derivative that has garnered significant interest in recent years due to its exceptional features, such as its small particle size and high surface area (≈2600 m^2^/g^−1^) [13]. Its structure consists of multiple layers of a wrinkled two-dimensional carbon sheet with oxygen functional groups, such as hydroxyl (–OH) and epoxy (–O–), located on the basal plane, with carbonyl (C=O) and carboxyl (–COOH) groups distributed at the sheet edges [14]. The oxygen functional groups expand the distance between their layers, making it a hydrophilic nanomaterial that disperses easily in aqueous solutions [15].

GO has potential applications in cement-based materials. Previous research has indicated that GO can affect the hydration process of cement [16,17,18]. Yet the underlying mechanisms are still ambiguous. Consequently, researchers have recently investigated the use of GO as a reinforcing agent for enhancing mechanical properties, which depend largely on the content and morphology of hydration products. Tong et al. [19] stated that the high specific surface area and high accessibility to oxygen functional groups of GO increase the calcium silica hydrate (C–S–H) density, decrease the microstructure’s porosity, and stabilise the composites. Likewise, according to Gong et al. [20], adding 0.03% GO to cement paste enhanced the non-evaporable water and calcium hydroxide content, resulting in a greater degree of hydration. Moreover, porosity decreases by 13.5% relative to the control sample due to enhanced hydration. In the same framework. Lin et al. [21] suggested that the oxygen functional groups in GO play a critical role in catalysing the cement hydration process by serving as adsorption sites for both water and cement components, as well as nucleation sites for cement hydration products.

The study conducted by Wang et al. [22] found that GO did not stimulate the hydration process or produce more hydration products, but its effect was clearer on the cement composite microstructure and the morphology of hydration products, especially calcium hydroxide (CH). Moreover, Yang et al. [23] reported via SEM characterisation of GO-modified cement paste that GO sheet surfaces were completely covered by hydration products, indicating the formation of a strong bond between GO sheets and the hydration products, resulting in an increase in compressive strength. In another study, through SEM examination. Li et al. [24] stated that adding GPC (graphene oxide polycarboxylate superplasticizer) to the cement matrix promoted the compact structure of the hydration products, limiting the development of micropores and enhancing mechanical properties. This study found that adding GPC decreased the crystallite size of calcium hydroxide (CH). Furthermore, Lv et al. [2,25,26,27,28] reported the mechanism of GO nanosheets at different dosages (0.01–0.06%) in controlling the microstructure of cement pastes: GO nanosheets significantly impacted cement microstructure, controlling the morphology of the hydration products (C–S–H, CH, Aft, and AFm). Additionally, this study reported the role of GO nanosheets in forming flower-like crystals, which make the microstructure denser and thus improve mechanical properties.

Despite the growing body of evidence supporting GO’s beneficial effects on cementitious composites, several aspects remain insufficiently characterised. In particular, the dose-dependent behaviour of GO across a wide concentration range spanning three orders of magnitude and its simultaneous influence on hydration kinetics, CH crystal morphology, microstructure, and long-term mechanical performance have not been systematically addressed in a single study. Furthermore, the transition mechanism by which GO shifts from acting as a hydration retarder at early ages to a hydration promoter at later ages remains unclear. Based on this background, the present study addresses these gaps by examining the effect of GO on cement paste and mortar at different dosages (0, 0.0005%, 0.005%, and 0.05% by weight of cement) and curing ages. The content of hydration products (C–S–H and CH) was evaluated by thermogravimetric (TGA) analysis, the morphology and crystal size of hydration products were characterised by X-ray diffraction (XRD) and scanning electron microscopy (SEM/EDS), and the criteria for improving mechanical performance were established through compressive and flexural strength testing of GO-modified cement mortars over a 90-day curing period.

## 2. Materials and Methods

### 2.1. Materials

**Figure 1:**
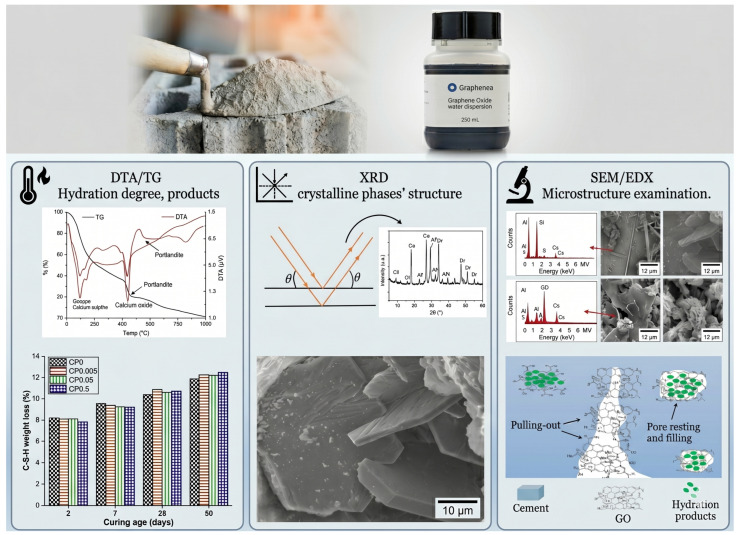
Structural schematic diagram of hydration process and microstructural changes in cement with GO. (**left**) DTA/TG analysis of hydration degree and products; (**centre**) XRD analysis of crystalline phases; (**right**) SEM/EDS examination of the microstructure.

To research the impact of varied GO doses in cementitious materials, this study employed cement paste and mortars prepared in accordance with the EN 196-1:2018 standard [29]. The chemical composition of the high initial strength Portland cement type CEM I 52.5 R used for this purpose is presented in Table 1, supplied by Holcim, Madrid, Spain. The GO used in this study was supplied by Graphenea Inc., San Sebastián, Spain as an aqueous dispersion with a concentration of 4 g/L, and its chemical composition is reported in Table 2. CEN-NORMSAND standardised sand adhering to the EN 196-1:2018 standard [29] was also used in cement mortars and procured in bags containing 1350 ± 5 g, Normensand was supplied by GmbH, Beckum, Germany. Figure 1 illustrates the conceptual framework of the hydration process and the expected microstructural evolution in cement composites incorporating GO.

As commercially available, GO typically consists of oxidised graphenic layers stacked with interlayer spacings expanded by oxygen functional groups, including hydroxyl (–OH) and epoxy (–O–) groups on the basal plane and carbonyl (C=O) and carboxyl (–COOH) groups at the sheet edges. It is acknowledged that strictly monolayered graphene is a distinct material [30]. According to the supplier’s characterisation data (Graphenea Inc., San Sebastián, Spain) [30], FTIR analysis confirms the presence of the following oxygen-containing functional groups: O–H stretch (3625 cm^−1^), C=O stretch (1772 cm^−1^), C=C (1644 cm^−1^), C–O (1380 cm^−1^), and C–O–C epoxy stretch (1069 cm^−1^). XPS quantification confirms that epoxide groups (C–O–C, 49.62%) represent the dominant contribution, followed by C sp3 (31.61%), C=O (5.01%), C–OH (1.67%), and O–C=O (0.76%). ^13^C solid-state NMR confirms sp2 hybridisation (130 ppm), C–OH (70 ppm), and C–O–C epoxy groups (60 ppm). XRD analysis reveals the (001) reflection at 2θ = 10.5°, corresponding to an interplanar spacing of ~9 Å, confirming successful exfoliation and interlayer expansion due to oxygen functional groups. The particle size distribution determined by laser diffraction is D10 = 6–7 µm, D50 = 14–17 µm, and D90 = 29–33 µm. AFM measurements confirm a flake thickness of approximately 1 nm, consistent with a monolayer or few-layer structure. The Raman characterisation of the GO was reported in a previous study by the authors [31], showing the characteristic D band (~1350 cm^−1^) and G band (~1580 cm^−1^), with a high D/G intensity ratio consistent with a highly defective oxidised graphenic structure.

**Table 1:** Chemical composition (wt.%) of Portland cement CEM I 52.5 R.

CEM I	Chemical Composition (wt.%)							
	CaO	SiO_2_	Al_2_O_3_	SO_3_	Fe_2_O_3_	MgO	K_2_O	LOI ^1^
**%**	61.5	20.5	5.03	3.35	3.20	1.45	1.05	2.39

^1^ Loss on ignition. Data adapted from [31,32].

**Table 2:** Chemical composition of GO (%).

Element	Carbon	Oxygen	Sulfur	Hydrogen	Nitrogen
**(%)**	49–56	41–50	0–2	0–1	0–1

Data adapted from [31,32].

### 2.2. Preparation of GO-Water Suspension

**Figure 2:**
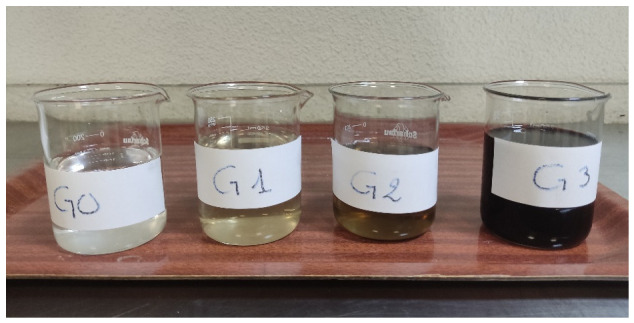
GO-water suspension prepared.

In this study, three GO dosages were used (0.0005%, 0.005%, and 0.05% by weight of cement). A reference paste and mortar mix was made without GO (0 wt.% GO) for comparison (Figure 2 shows the prepared solutions). The mixing process of GO in water was as follows: the GO solution volume corresponding to the dose’s weight was pipetted into a pipette and then poured into the distilled water (ensuring that the total water content adhered to the w/c ratio of 0.5, with the volume of water present in the GO solution being subtracted accordingly). Next, the solution was stirred with a magnetic stirrer for 5 min at a rotational speed of 800 rpm.

After stirring, pH and electrical conductivity (EC) were measured. The initial pH of distilled water (5.03) decreased with increasing GO content, reaching 3.93 at 0.05% GO, due to the presence of acidic oxygen-containing functional groups. Electrical conductivity increased from 1.36 µS/cm to 42.1 µS/cm, indicating the introduction of charged species from the GO dispersion.

The selected dosages span three orders of magnitude to systematically evaluate the dose-dependent effects of GO on hydration, microstructure, and mechanical performance, in line with dosage ranges reported in the literature [2,25,26,27,28].

### 2.3. Preparation of Cement Paste and Mortar

In the present study, two kinds of cement composites were prepared. Cement paste samples were prepared for thermogravimetric analysis (TGA), X-ray diffraction (XRD), and scanning electron microscopy (SEM). In parallel, cement mortar samples were prepared for mechanical properties, such as compressive and flexural strengths.

Once the GO-water solution had been prepared, the dose was put into the automatic rotary mixer bowl with the cement required (Table 3 and Table 4 show the proportions of the GO-modified cement paste and mortar mixtures, keeping a constant 0.5 w/c).

In the case of cement paste, the mixing process starts at a low speed and lasts 90 s. Then, after a 30 s pause for a break, the mixture is mixed again at low speed for 90 s.

As for cement mortar, the samples were prepared under European Standard EN 196-1:2018 [29]. The prepared GO-water solution was put into a bowl of the automatic rotary mixer with the required cement. The mixing process began at a slow rate for 30 s, followed by a consistent addition of sand for a further 30 s, then a 90 s pause, after which the mixing process recommenced at a rapid pace for 60 s.

After completion of the mixing process, two types of prismatic molds were used: one with dimensions of (10 × 10 × 60 mm^3^) for cement paste and another with dimensions of (40 × 40 × 160 mm^3^) for cement mortar. The mixture was divided into two layers and compacted. Each layer received 60 strokes to remove any air bubbles. Then, the mold surface was covered with plastic sheets to prevent water loss, and the molds were stored in the curing room (20 ± 2 °C/RH > 95%). After 24 h, all samples were de-molded, labelled, and returned to the curing room until the test day.

### 2.4. Characterisation Techniques

#### 2.4.1. Thermal Gravimetric Analysis (TGA)

This technique was used to study the effect of GO on the hydration product contents in cement paste. The samples (CP0, CP0005, CP005, and CP05) were subjected to a hydration stopping process before being crushed into powder on the test day. Hydration was halted by first drying the specimens under vacuum for 30 min, followed by immersion in isopropanol for 24 h. The prepared powders were subjected to quantitative thermogravimetric analysis (TGA) using LABSYS Evo equipment (SETARAM Instrumentation, Caluire, France) within a temperature range of 20 °C to 1100 °C, at a heating rate of 10 °C/min under a dynamic N_2_ atmosphere. Thermogravimetry differential scanning calorimetry (TG-DSC) curves were used to calculate sample weight losses, allowing the assessment of GO’s influence on the hydration degree of cement paste.

#### 2.4.2. X-Ray Diffraction (XRD)

X-Ray Diffraction (XRD) was employed to characterise the phases and crystal size of the hydration products of GO-modified cement paste samples (CP0, CP0005, CP005, and CP05) at 28 days of curing using a Bruker model D8 ADVANCE diffractometer with Cu Kα radiation (λ = 1.54060 Ǻ). The specimen was ground to a powder, and 10–15 g were collected to be sifted through a 53 μm sieve. Powder samples were scanned from 2 to 65° 2θ with a step size of 0.02° 2θ and a counting time of 1 s for each step.

#### 2.4.3. Scanning Electron Microscope and Energy Dispersive X-Ray Spectroscopy (SEM/EDS)

Microstructural GO-modified cement paste (CP0, CP0005, CP005, and CP05) was characterised by Scanning Electron Microscope and Energy Dispersive X-ray Spectroscopy (SEM/EDS, JEOL JSM-820). After 28 days of curing, the hydration was stopped. The samples were cut from the centre into thin square sections, mounted on the sample substrate, and then coated with gold prior to characterisation.

#### 2.4.4. Macro-Mechanical Strength

The mechanical properties were evaluated on the cement mortar prism specimens (CM0, CM0005, CM005, and CM05) at 2, 7, 28, and 90 days in two ways, following the procedure in compliance with the standard EN 196-1:2018 [29]. The flexural strength tests were carried out using a three-point bonding machine (IberTest C1B1400, Madrid, Spain). The average flexural strength was obtained after testing 3 specimens for each type. Compressive strength tests were conducted on the two halves of each specimen resulting from the preceding flexural test using a hydraulic machine (IberTest model HIB 150).

## 3. Results and Discussion

### 3.1. Thermal Gravimetric Analysis (TGA) Results

Differential thermal and thermogravimetric tests (DTA-TG) were performed according to ASTM E 1131 [33] to determine the effect of GO on the content of hydration products, such as C–S–H gel and CH. As is well recognised, the content of hydration products, particularly (C–S–H) gel, plays a significant role in defining the strength and cohesion of cementitious materials [34].

**Figure 3:**
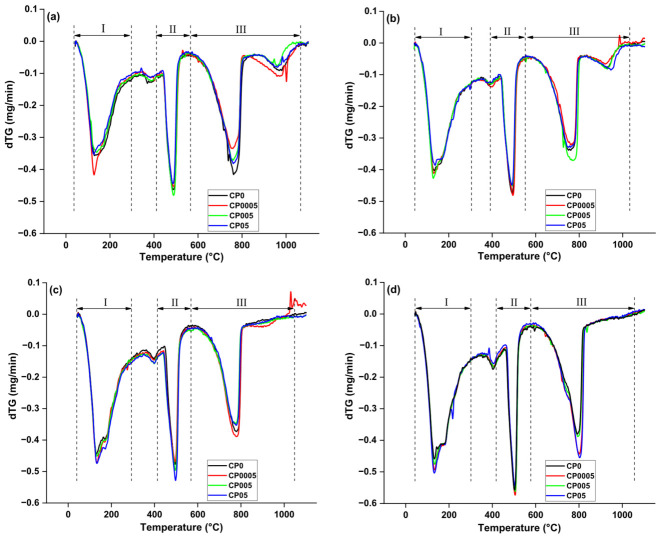
Curves of thermal analysis (dTG vs. temperature) of cement paste with different GO dosages at (**a**) 2 days; (**b**) 7 days; (**c**) 28 days; (**d**) 90 days. Data adapted from [32].

**Figure 4:**
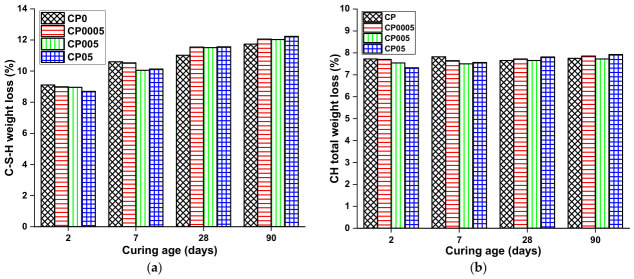
Weight loss of (**a**) C–S–H; and (**b**) CH, at different curing ages. Data adapted from [32].

Figure 3 shows the TG-DTA results of the different paste samples CP0, CP0005, and CP05, at the ages of 2, 7, 28, and 90 days. All samples have three prominent peaks. The first endothermic peak (I) is at around 110–300 °C, corresponding to the weight loss of water from C–S–H and Aft, corresponding to the dehydration of C–S–H and ettringite (AFt) (tag: C–S–H_Loss). The second (II) peak is also endothermic and occurs at around 430–530 °C, associated with the dehydroxylation of portlandite CH(tag: CH_Loss). The third peak (III) from 550 °C to 1000 °C is attributed to the decomposition of calcium carbonate (CaCO_3_) (tag: CO_2__Loss) [35]. Furthermore, CH_Loss total additionally considers the water loss resulting from the carbonation of portlandite as CH_Total.Loss = CH_Loss + 0.41 CO_2__Loss [36]. The values of weight loss, C–S–H gel, and CH, which are presented in Figure 4, were computed using the data displayed in Figure 3.

According to stages I and II, corresponding to the dehydration of C–S–H gel and CH dihydroxylation, GO did not affect the C–S–H gel and CH content produced during cement hydration at an early age. At 2 and 7 days, the C–S–H gel and CH content are greater in the reference sample CP0 compared with the samples containing GO. Furthermore, when comparing samples containing different doses of GO, it can be seen that as the GO dose increases, the amount of both C–S–H gel and CH produced decreases. This can be attributed to GO’s high specific surface area and hydrophilic nature, where the larger the GO dose, the greater the amount of water absorbed. GO layers are interconnected by oxygen functional groups, allowing GO to absorb water between the layers. Researchers [37,38,39] have demonstrated that inserting oxygen functional groups into GO increases the interlayer spacing distances, enhancing its absorption capacity. Thus, consideration must be given to reducing the effective water-to-cement ratio. Since cement paste containing GO had less water, certain cement particles did not fully react and remained unhydrated. This resulted in lower C–S–H gel and CH content in GO-containing cement compared with the reference sample CP0 at an early age (2 and 7 days). The study of Wang et al. [40] revealed that the incorporation of GO at various doses into cement containing a stable amount of fly ash did not increase the hydration degree at an early age but rather somewhat inhibited the content of hydration products. Similarly, Li et al. [16] reported that GO did not affect the mechanical properties of cement at an early age due to the low hydration degree of cement containing GO. They asserted that GO absorbed part of the mixing water, leaving less water available for cement hydration. Additionally, research by Li et al. [41] demonstrated that incorporating GO into fresh cement paste instantly decreases the concentration of calcium cations owing to their strong chemical cross-linking with GO. A reduction in the concentration of Ca^2+^ might retard hydration.

Nonetheless, after 28 days, the reverse of the early-age outcomes was seen. Stage I, which corresponds to the dehydration of C–S–H, revealed that different GO dosages affected the C–S–H weight loss. Compared with the reference sample CP0, samples CP0005, CP005, and CP05 displayed greater C–S–H weight-loss increases after 28 days by about 4.82%, 4.45%, and 4.90%, respectively. At 90 days, the C–S–H weight loss for samples CP0005, CP005, and CP05 is estimated to be higher by 2.81%, 2.15%, and 4.25%, respectively, compared with the control sample CP0. Regarding stage (II), which corresponds to the decomposition of CH, it can be seen that the effect of GO is slight. The weight loss of CH for samples containing GO was comparable with that of the standard sample CP0. However, the greatest CH weight loss was observed in sample CP05, with values of 3.83% on day 28 and 3.92% on day 90 compared with the reference sample.

The hydrophilic nature of GO and the presence of water between its layers may account for the long-term hydration observed. The high specific surface area and oxygen functional groups of GO allow it to be a hydrophilic nanomaterial, making it a water storage centre, absorbing the free water present in cement paste at the initial moment of mixing and inhibiting the rapid evaporation of the retained free water. With an uninterrupted hydration process, when the free water in the internal cement system is inadequate for sustaining the hydration process, GO will progressively release the free water adsorbed between layers to constantly promote hydration over time and ensure its continuity [42,43]. As a result, the content of hydration products, especially C–S–H gels, increases with age. As discussed above, Li et al. [16] reported that the hydration degree improved over time because of the gradual release of absorbed water. In the same sense, Lin et al. [21] found that the catalytic behaviour of GO accelerated the hydration rate, with the water molecules on GO constituting a water reservoir and thus water transport channels for the hydration of C_3_S, C_2_S, C_4_AF, and C_3_A.

It is also hypothesised that hydration occurs directly on the GO layer’s surface, characterised by a high surface area and acting as a nucleation site. Where the adsorption of GO’s oxygen functional groups to cement particles facilitates the occurrence of the hydration process on the surface of the GO layers. It has been estimated that the interlayer spacing distance between the GO layers is ~6–7 nm [44], which could lead to the potential formation of C–S–H gel, ultimately distributed uniformly within.

At 28 and 90 days, the cement paste containing 0.05% GO (CP05) exhibits a well-defined peak of calcium carbonate (CaCO_3_) decomposition, as shown in Figure 3. This observation suggests that GO plays a significant role in modifying the carbonation process in a way that enhances the durability of the cement matrix. Several studies have reported that GO enhances the formation of a denser and more compact microstructure due to its high surface area and functionalised oxygen groups, which facilitate nucleation and growth of hydration products. This densification effect can contribute to a controlled and beneficial formation of CaCO_3_ rather than excessive carbonation that weakens the structure.

GO’s ability to regulate moisture retention and promote hydration kinetics results in a matrix with fewer capillary pores, reducing permeability to CO_2_ ingress. Previous studies have confirmed that GO improves the resistance of cement-based materials to aggressive environmental exposure, including carbonation and chloride penetration [44]. The gradual formation of CaCO_3_ at later curing stages (28 and 90 days) can be linked to the delayed release of water stored within GO interlayers, which sustains continued hydration and enhances the long-term stability of the cementitious matrix.

Thus, the presence of GO in CP05 not only enhances hydration at later ages but also contributes to improving resistance to early carbonation effects by promoting a more compact and durable cement structure. These findings align with previous research emphasising the long-term benefits of GO in cementitious systems, demonstrating that it serves as an effective durability-enhancing additive that strengthens the microstructure and limits unwanted carbonation.

To conclude, in the later (advanced) stages of hydration, the greater weight loss of C–S–H and CH in GO-modified samples compared with the reference sample illustrates the positive impact of GO on the cement hydration process. A possible outcome of this approach is an improvement in the mechanical properties of GO-modified cement composites at advanced ages.

### 3.2. X-Ray Diffraction (XRD) Results

Previous thermogravimetric analysis (TGA) results showed that GO influenced the hydration product content only slightly, particularly for CH. Nevertheless, aside from the CH content, the morphology of the CH crystals in the cement paste composite, such as shape and size, was found to significantly affect its performance. It is theorised that the smaller the CH crystal size in cement, the stronger the adhesion strength to the (C–S–H) gel, which can improve the microstructure formation and performance of the cement composite [24,40].

**Figure 5:**
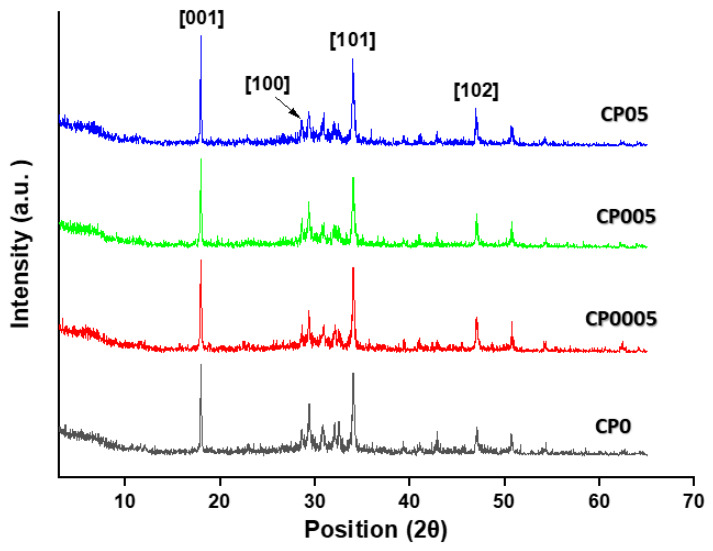
XRD patterns of the cement paste sample (CP0, CP0005, CP005, and CP05) at 28 days of curing. Data adapted from [32].

XRD was utilised to determine the grain size of CH crystals to comprehend the effect of different GO doses on CH crystal size in cement paste. As shown in Figure 5, the main reflections of CH crystal planes [001], [100], [101], and [102] are situated close to 18°, 28°, 34°, and 47°, respectively. The size of the CH crystals was calculated using the Scherrer equation [45], which is given by the following formula:

$$D=\frac{K\cdot \lambda }{\beta \cdot cos\theta }$$

where

*D* is the crystal grain size in the direction perpendicular to the crystal plane (nm);

*K* is Scherrer constant, which is 0.89;

*λ* is the wavelength of X-ray, which is 0.15 nm;

*β* is the diffraction peak half-width;

*θ* is the diffraction angle.

The statistical size of CH crystals was determined by calculating the average grain size from multiple diffraction peaks, ensuring accuracy and consistency across all samples. At least four independent measurements were performed per peak, and the mean value was obtained to provide a representative CH grain size distribution. This method ensures statistical reliability and allows for a meaningful comparison between different GO dosages.

Figure 5 presents the XRD patterns of cement paste samples CP0, CP0005, CP005, and CP05 after 28 days of curing. It can be seen that the position of the peaks did not vary. However, the intensity exhibited some variation. This suggests that the cement hydration crystals of all samples had the same crystalline phase with different amounts. This was further corroborated by the results obtained from thermogravimetric analysis (TGA).

Table 5 displays the grain size of CH crystals and the reduction percentage at each diffraction peak for each sample. As can be observed, GO has a substantial effect on lowering the grain size of CH crystals. Virtually all of the samples showed a drop in CH crystal grain size. However, as always, the sample CP05, which contains 0.05% GO, showed the highest decrease in CH crystal grain size and became more refined, with an estimated decrease in planes of diffraction peaks [001], [100], [101], and [102] of 13.45%, 8.90%, 8.27%, and 15.43%, respectively. This significant reduction in CH crystal grain size is evidence that adding 0.05% GO to the cement paste system drives CH crystals to convert into crystalline states different from the hexagonal crystal. This is what can be observed in SEM images.

The results revealed a significant reduction in the CH crystal size in sample CP05, which had the highest CH content according to the previous TGA results. Thus, as the amount of CH crystal grains rises, their size tends to decrease. The introduction of GO appears to interact with CH during its nucleation and growth phases, potentially restricting the crystallite growth. This interaction implies that, as CH begins to form and integrates with GO, its size remains limited rather than expanding significantly. Thus, the incorporation of GO likely leads to a smaller CH crystal formation. Thus, enhancing adhesion to other hydration products, such as C–S–H gel and forming a finer microstructure, which contributes more to the strength improvement. This phenomenon has also been observed in previous studies [22,24,40].

The reduction in CH crystal size measured in this study corresponds to the grain size calculated perpendicular to the crystallographic planes analysed via XRD. The Scherrer equation provides an indirect measurement of crystal size based on diffraction broadening rather than direct geometric dimensions, such as length or width, observed in SEM images. However, previous studies have shown that a reduction in XRD-derived grain size correlates well with the overall decrease in CH particle dimensions, contributing to a more refined and compact microstructure.

### 3.3. Microstructural and Elemental Analysis

**Figure 6:**
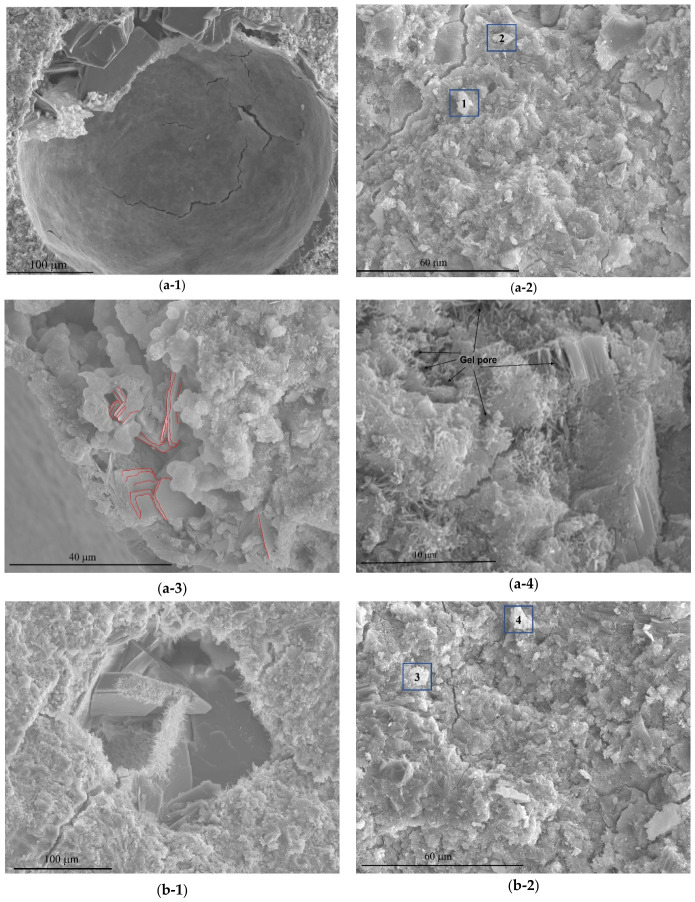

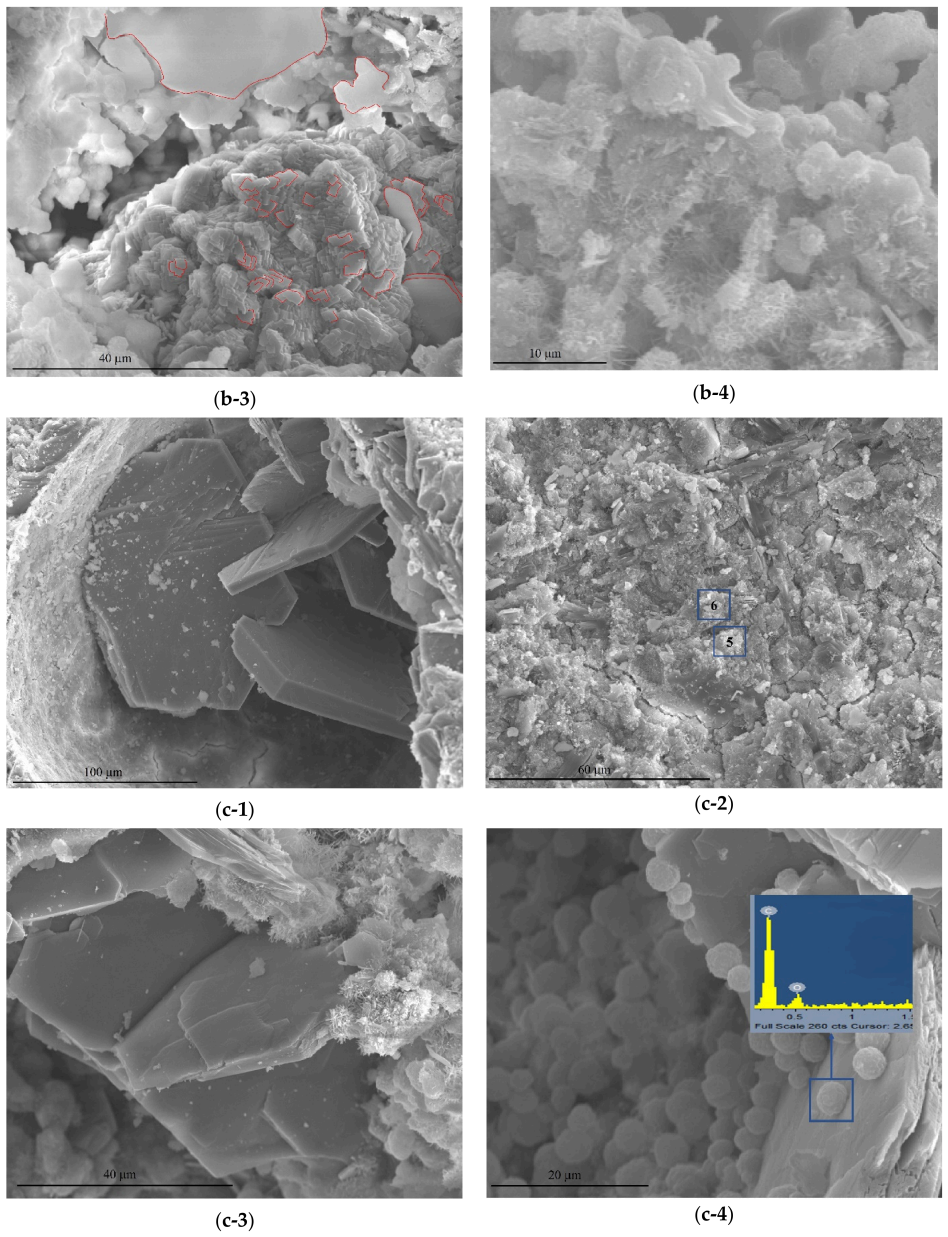

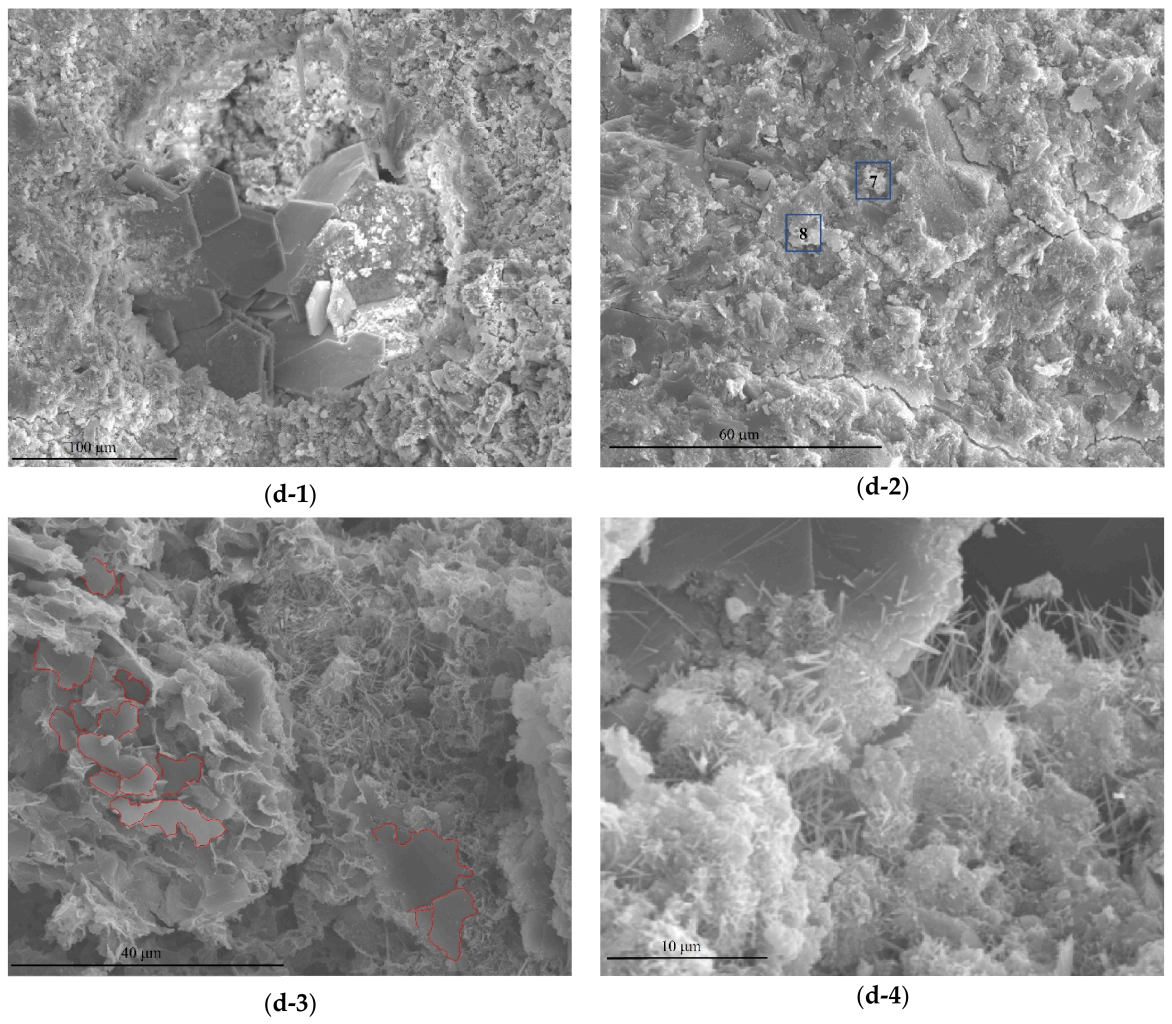
Microstructure of the investigated samples after 28 days of hydration, (**a**) CP0, (**b**) CP0005, (**c**) CP005 and (**d**) CP05. Data adapted from [32]. Columns: (-1) pores; (-2) microcracks; (-3) CH crystals; (-4) C–S–H gel and ettringite.

Scanning electron microscopy (SEM) was employed to examine the microstructural features of the cement paste samples with different graphene oxide contents (0.0005, 0.005, and 0.05% GO). Figure 6 shows the SEM images of the samples at four magnification levels. The microstructure of each sample was compared with that of the reference sample (0% GO) to evaluate the effect of GO on the morphology of hydration products. The microstructure of all samples was compared following the scale from highest to lowest.

In Figure 6(a-1), it was observed that the reference sample CP0 had a pore of 348 μm in diameter that seemed almost empty. This is observed in the majority of the pores of this sample. In contrast, the GO-containing samples have smaller pores than the reference sample. In Figure 6(d-1), the pore diameter of CP05 is 181 μm, and the pore diameter of CP0005 (b-1) is estimated to be 192 μm. The pores are almost filled with hydration products, mostly with calcium hydroxide (CH). This indicates the pore-filling effect of GO. Furthermore, as seen in Figure 6(a-2,b-2,c-2,d-2), all samples had cracks of varying width and continuity in their microstructure. The microstructure of CP0 has wider cracks with continuity than the GO-containing samples, which have narrow and discontinuous cracks.

The previous XRD analyses indicated that incorporating GO tended to decrease the grain size of CH crystals. To validate this trend, the statistical size of CH was also measured through SEM images. While XRD and SEM represent distinct methods. XRD provides crystallite size within grains, and SEM captures grain size composed of multiple crystals, the measurements from both techniques show a similar trend in size reduction with GO incorporation. Generally, the crystallite sizes obtained through XRD are smaller than those measured by SEM, due to differences in detection depth and field of view, with SEM providing a more extensive view of the microstructure. Nonetheless, the results from both methods are comparable within their respective methodological limits, collectively reinforcing the observed trend of reduced CH crystal size with GO addition.

Red lines were added to the images (a-3), (b-3), and (d-3) in Figure 6 to highlight the CH crystals’ morphology and dimensions more clearly. The addition of GO to the cement paste dramatically alters the CH crystals’ morphology compared with the reference sample. Figure 6(a-3) display the lamellar, hexagonal, and parallel structure of the CH crystals in CP0. Additionally, Figure 6(b-3,d-3) reveal that the CH crystals in CP0005 and CP05 have completely lost their hexagonal structure, are more coherently bonded together, and have edges that have been eroded. The statistical size of the CH crystal for CP0 is 7.84 μm, whereas the statistical sizes for CP0005 and CP05 are 2.18 μm and 5.04 μm, respectively. The morphology and size of CH crystals might be affected by the presence of GO. This could be due to GO serving as a nucleation site for the hydration products, including CH crystals, and/or the functional oxygen groups on graphene oxide’s surface, such as C–OOH or C–OH reactivity with CH crystals, resulting in its decomposition [17,18]. This results in smaller and more distributed particles, which would explain the observed alterations. Except for the microstructure of sample CP005, we could not identify an insufficient number of CH crystals. Nonetheless, the number observed in certain areas appears to be greater even than that of the reference sample. The explanation seems clear, suggesting that this dose (0.005%) remained spherical agglomeration (Figure 6(c-4)) that hindered the nucleation of CH crystals on their surface and prevented any interaction.

Lastly, the distribution and density of the C–S–H gel were also highlighted. Figure 6(a-4) of sample CP0 reveal that the C–S–H gel is poorly dense and disordered, entangled with CH crystals. Additionally, the microstructure of CP0 contains ettringite crystals (AFt) that are irregularly distributed among the C–S–H gel in thick needle-like and rod-like shapes.

Furthermore, the matrix has numerous gel pores (as indicated in Figure 6(a-4)) and cracks that reduce the cohesion and homogeneity of the C–S–H gel, resulting in voids and defects in the microstructure. In addition, Figure 6(b-4,d-4) of samples CP0005 and CP05, respectively, show that the C–S–H gel is denser and more homogeneous, covering the edges of CH crystals effectively with fewer gel pores. However, these samples have more abundant but thinner ettringite crystals, a phenomenon also observed in previous work by S.M. Monteagudo et al. [46] with the incorporation of nano-silica, which similarly led to thinner ettringite formations. This suggests a comparable mechanism at play with GO and nano-silica additives, promoting the refinement of ettringite crystal morphology.

To examine the effect of GO on the microstructure compositions of the cement paste, spots in the images (a-2), (b-2), (c-2), and (d-2) of Figure 6 were marked with blue squares to indicate the regions likely to contain gel (C–S–H) and the spots chosen were exposed to a high-energy X-ray beam (EDS). The results of the EDS analysis are shown in Table 6. Generally, the C–S–H gel consists of Ca, Si, and O, while the H atom is too light to be detected by EDS. In our case, trace amounts of Al and Fe were detected. This implies that the C–S–H gel is crosslinked with AFt/AFm and CH. As shown in Table 6, samples CP0005 and CP05 showed a higher proportion of oxygen and carbon in their C–S–H gel compared with the reference sample CP0. It is also noteworthy that sample CP005 had a markedly lower proportion of oxygen and carbon, likely due to the poor dispersion of this dosage (0.005%), which remains in the shape of small spheres, as seen in the preceding image (C-4) in Figure 6. The samples CP0005 and CP05 displayed C–S–H gel compositions consisting of proportions of carbon and oxygen, confirming the GO acting as a nucleation site. This suggests that hydration products form and proliferate on the GO surface plane as a consequence of their water-holding capacity, a hypothesis previously proposed in TGA results. Interestingly, the pure state of GO incorporated in samples CP0005 and CP05 could not be determined. The formation of strong covalent bonds between GO oxygen functional groups, such as carboxylic acid (–COOH), and hydration products C–S–H and CH on the GO plane surface makes it difficult to establish the pure state of GO in the cement matrix. Previous studies [23,47,48] have reported the formation of hybrid materials via chemical interactions between GO’s carboxylic acid groups and the hydration products C–S–H and CH. In contrast, GO in sample CP005 was easily recognised physically and identified chemically, indicating that this dose failed to serve as a nucleation site. As a result, carbon and oxygen were not detected in this sample’s C–S–H gel. The results obtained in this experiment are congruent with those of prior TGA and XRD studies, which suggested that samples CP0005 and CP05, containing respective doses of 0.0005% and 0.05% GO, demonstrate greater efficacy due to their nucleation site, compared with reference sample CP0, and even in comparison to sample CP005. CP0005 and CP05 samples exhibited high contents of hydration products, such as C–S–H gel, and CH produced at an advanced age, which similarly showed their effectiveness in diminishing CH crystal sizes.

### 3.4. Mechanical Strength Results

Following nanoscale research of the impact of GO on cement paste, the attention was shifted to the impact of GO on cement mortar, focusing on mechanical properties, such as compressive and flexural strength, as the macro-properties results are inextricably linked to the nanoscale results.

**Figure 7:**
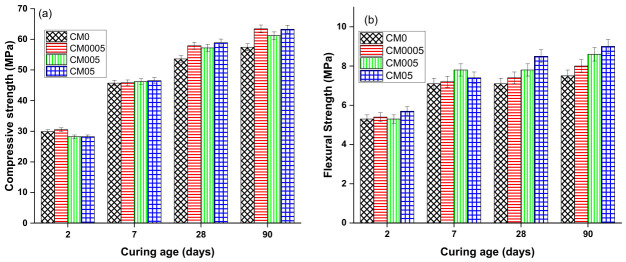
Mechanical properties (**a**) The compression and (**b**) flexural strength of mortar with different dosages of GO and at different curing ages.

The average results of compressive and flexural strengths of mortar samples CM0, CM0005, and CM05 as a function of curing time (2, 7, 28, and 90 days) are shown in Figure 7. At an early age (2 and 7 days), the compressive strength of the tested mortar specimens did not increase when varying doses of GO were added. The reference sample had higher compressive strength than the specimens with GO. Furthermore, specimen CM05, with the greatest dose of GO (0.05%), has the lowest compressive strength. This can be attributed to water absorption lessening the amount of hydration products formed in the hydration process, which are known to be the main determinants of the mechanical properties of cement composites, especially C–S–H gel (This is based on the assumption that water absorption increases with higher GO doses). In contrast to the compressive strength, the flexural strength of GO-containing mortar specimens increased somewhat at an early age. Nonetheless, the divergence from the reference specimen was not substantial. The thermogravimetric analysis results discussed above support this conclusion.

The results at 28 and 90 days exhibited a distinct pattern from those at early ages. The compressive strength of cement mortar specimens containing GO was found to be greater than that of the reference specimen CM0. Notably, specimens CM0005 and CM05, which contained GO doses of 0.0005% and 0.05%, respectively, exhibited the most significant improvement in compressive strength. CM0005 showed an increase in compressive strength of 7.43% and 9.46% at 28 and 90 days, respectively, while specimen CM05 showed an increase of 9.00% and 9.32% at these same time points relative to CM0.

With respect to flexural strength, the results reveal that flexural strength rises as the amount of GO added increases. The flexural strength of the specimens CM0005 and CM05 improved by 4.06% and 16.47% after 28 days and by 6.25% and 16.67% after 90 days, relative to CM0.

This research has shown that a variety of factors can enhance the mechanical strength of cement composites. Graphene oxide (GO) acts as a nucleation site that facilitates the hydration process and increases the density of the C–S–H gel. Numerous studies [10,16,49] have validated the effect of GO as a nucleation site on the mechanical strength of cement composites. Moreover, the acceleration of the hydration process gives rise to the formation of more hydration products, which makes the microstructure denser with fewer cracks and filled pores. Additionally, it has been suggested that the morphology of CH crystals is also beneficial for raising mechanical strength. XRD and SEM analyses have revealed that GO decreases the size of CH crystal and makes it finer. Several studies [22,40,42] commonly accept that higher mechanical strength is related to smaller crystal sizes.

## 4. Conclusions

The DTA results indicated that GO accelerated the hydration process at later ages. However, the early age improvement with GO was constrained by the retarding effect of the GO that absorbed water initially and acted as a water storage centre.

The XRD results indicated that the incorporation of GO reduced CH crystal size and enhanced the density of the cementitious matrix. This trend was also observed in the SEM analysis, which supported the reduction in grain size with GO addition. Specifically, the sample CP05 (with 0.05% GO) exhibited the smallest CH crystals after 28 days of curing, highlighting the consistent effect of GO across both XRD and SEM observations.

SEM images reveal the efficiency of GO in densifying the microstructure, narrowing cracks, and preventing their propagation, as well as its obvious influence on pore filling.

The mechanical strength analysis showed that GO had a greater effect at later ages. The optimal dosages were found to be 0.0005 and 0.05%, which enhanced the strength through various reinforcing mechanisms of GO, such as increasing the hydration degree by acting as a nucleation site, refining the CH crystals, and densifying the microstructure.

## Figures and Tables

**Table 3:** Cement paste mix proportions.

Mix Proportion	Cement (g)	w/c	Water Content (mL)			GO (%)	GO (mg)
			Distilled Water	GO Dispersion 4 g/L	Total Water		
CP0	450	0.5	225	0	225	0	0
CP0005	450	0.5	224.438	0.5625	225	0.0005	2.25
CP005	450	0.5	219.375	5.625	225	0.005	22.50
CP05	450	0.5	168.750	56.25	225	0.05	225

**Table 4:** Cement mortar mix proportions.

Mix Proportion	Cement (g)	Sand (g)	w/c	Water Content (mL)			GO (%)	GO (mg)
				Distilled Water	GO Dispersion 4 g/L	Total Water		
CM0	450	1350	0.5	225	0	225	0	0
CM0005	450	1350	0.5	224.438	0.5625	225	0.0005	2.25
CM005	450	1350	0.5	219.375	5.625	225	0.005	22.5
CM05	450	1350	0.5	168.750	56.25	225	0.05	225

**Table 5:** The planes of diffraction peak of CH crystal and their size at 28 days. Data adapted from [32].

Samples	[001]		[100]		[101]		[102]	
	Crystal Size (nm)	Decrease * (%)	Crystal Size (nm)	Decrease * (%)	Crystal Size (nm)	Decrease * (%)	Crystal Size (nm)	Decrease * (%)
CP0	59.63	--	48.90	--	34.84	--	32.25	--
CP0005	53.97	10.49	45.87	6.60	33.61	3.68	29.93	7.76
CP005	53.35	11.78	49.27	−0.75	33.01	5.57	33.27	−3.07
CP05	52.56	13.45	44.90	8.90	32.18	8.27	27.94	15.43

* In relation to CP0.

**Table 6:** The chemical elements of the points shown in Figure 6. Data adapted from [32].

Samples	Elements	Ca	Si	Al	Fe	O	C
		Weight Percentage (%)					
CP0	Pointe 1	54.22	14.83	2.01	--	28.94	--
	Pointe 2	48.60	10.16	3.47	5.68	32.10	--
CP0005	Pointe 3	44.35	13.46	0.55	--	30.27	11.37
	Pointe 4	31.83	8.48	--	--	48.21	11.48
CP005	Pointe 5	36.65	11.19	1.78	--	50.38	--
	Pointe 6	41.84	10.75	2.44	--	39.10	5.87
CP05	Pointe 7	40.57	14.58	--	--	35.34	9.50
	Pointe 8	25.94	8.51	2.03	--	47.53	15.99

## Data Availability

The original contributions presented in this study are included in the article. Further inquiries can be directed to the corresponding author.

## References

[1] Pan Z., He L., Qiu L., Korayem A.H., Li G., Zhu J.W., Collins F., Li D., Duan W.H., Wang M.C. (2015). Mechanical properties and microstructure of a graphene oxide-cement composite. Cem. Concr. Compos..

[2] Lv S., Ma Y., Qiu C., Sun T., Liu J., Zhou Q. (2013). Effect of graphene oxide nanosheets of microstructure and mechanical properties of cement composites. Constr. Build. Mater..

[3] Peng H., Ge Y., Cai C.S., Zhang Y., Liu Z. (2019). Mechanical properties and microstructure of graphene oxide cement-based composites. Constr. Build. Mater..

[4] Adesina A. (2020). Nanomaterials in cementitious composites: Review of durability performance. J. Build. Pathol. Rehabil..

[5] Mohammed A., Sanjayan J.G., Duan W.H., Nazari A. (2015). Incorporating graphene oxide in cement composites: A study of transport properties. Constr. Build. Mater..

[6] Ng D.S., Paul S.C., Anggraini V., Kong S.Y., Qureshi T.S., Rodriguez C.R., Liu Q.F., Šavija B. (2020). Influence of SiO_2_, TiO_2_ and Fe_2_O_3_ nanoparticles on the properties of fly ash blended cement mortars. Constr. Build. Mater..

[7] Abhilash P.P., Nayak D.K., Sangoju B., Kumar R., Kumar V. (2021). Effect of nano-silica in concrete; A review. Constr. Build. Mater..

[8] Devasena M., Sangeetha V. (2021). Implications of Nano-Titanium Dioxide Incorporation in Cement Matrix: A Review. J. Inst. Eng. Ser. D.

[9] Qin R., Zhou A., Yu Z., Wang Q., Lau D. (2021). Role of carbon nanotube in reinforcing cementitious materials: An experimental and coarse-grained molecular dynamics study. Cem. Concr. Res..

[10] Anwar A., Liu X., Zhang L. (2023). Nano-cementitious composites modified with Graphene Oxide—A review. Thin-Walled Struct..

[11] Murali M., Alaloul W.S., Mohammed B.S., Musarat M.A., Al Salaheen M., Al-Sabaeei A.M., Isyaka A. (2022). Utilizing graphene oxide in cementitious composites: A systematic review. Case Stud. Constr. Mater..

[12] Bheel N., Ali M.O.A., Kırgız M.S., Shafiq N., Gobinath R. (2023). Effect of graphene oxide particle as nanomaterial in the production of engineered cementitious composites including superplasticizer, fly ash, and polyvinyl alcohol fiber. Mater. Today Proc..

[13] Suk J.W., Piner R.D., An J., Ruoff R.S. (2010). Mechanical properties of monolayer graphene oxide. ACS Nano.

[14] Maity I., Ghosh K., Rahaman H., Bhattacharyya P. (2017). Selectivity Tuning of Graphene Oxide Based Reliable Gas Sensor Devices by Tailoring the Oxygen Functional Groups: A DFT Study Based Approach. IEEE Trans. Device Mater. Reliab..

[15] Montes-Navajas P., Asenjo N.G., Santamaría R., Menéndez R., Corma A., García H. (2013). Surface area measurement of graphene oxide in aqueous solutions. Langmuir.

[16] Li X., Li C., Liu Y., Chen S.J., Wang C.M., Sanjayan J.G., Duan W.H. (2018). Improvement of mechanical properties by incorporating graphene oxide into cement mortar. Mech. Adv. Mater. Struct..

[17] Lu Z., Hou D., Meng L., Sun G., Lu C., Li Z. (2015). Mechanism of cement paste reinforced by graphene oxide/carbon nanotubes composites with enhanced mechanical properties. RSC Adv..

[18] Horszczaruk E., Mijowska E., Kalenczuk R.J., Aleksandrzak M., Mijowska S. (2015). Nanocomposite of cement/graphene oxide—Impact on hydration kinetics and Young’s modulus. Constr. Build. Mater..

[19] Tong T., Fan Z., Liu Q., Wang S., Tan S., Yu Q. (2016). Investigation of the effects of graphene and graphene oxide nanoplatelets on the micro- and macro-properties of cementitious materials. Constr. Build. Mater..

[20] Gong K., Pan Z., Korayem A.H., Qiu L., Li D., Collins F., Wang C.M., Duan W.H. (2015). Reinforcing Effects of Graphene Oxide on Portland Cement Paste. J. Mater. Civ. Eng..

[21] Lin C., Wei W., Hu Y.H. (2016). Catalytic behavior of graphene oxide for cement hydration process. J. Phys. Chem. Solids.

[22] Wang Q., Li S., Wang J., Pan S., Lv C., Cui X., Guo Z. (2018). Effect of Graphene Oxide on Hydration Process and Main Hydration Products of Cement. Kuei Suan Jen Hsueh Pao/J. Chin. Ceram. Soc..

[23] Yang H., Monasterio M., Cui H., Han N. (2017). Experimental study of the effects of graphene oxide on microstructure and properties of cement paste composite. Compos. Part A Appl. Sci. Manuf..

[24] Li Q., He C., Zhou H., Xie Z., Li D. (2021). Effects of polycarboxylate superplasticizer-modified graphene oxide on hydration characteristics and mechanical behavior of cement. Constr. Build. Mater..

[25] Lv S., Ma Y., Qiu C., Zhou Q. (2013). Regulation of go on cement hydration crystals and its toughening effect. Mag. Concr. Res..

[26] Lv S., Liu J., Sun T., Ma Y., Zhou Q. (2014). Effect of GO nanosheets on shapes of cement hydration crystals and their formation process. Constr. Build. Mater..

[27] Lv S., Ting S., Liu J., Zhou Q. (2014). Use of graphene oxide nanosheets to regulate the microstructure of hardened cement paste to increase its strength and toughness. CrystEngComm.

[28] Lv S.H., Deng L.J., Yang W.Q., Zhou Q.F., Cui Y.Y. (2016). Fabrication of polycarboxylate/graphene oxide nanosheet composites by copolymerization for reinforcing and toughening cement composites. Cem. Concr. Compos..

[29] (2018). Methods of Testing Cement: Part 1: Determination of Cement Strength.

[30] Graphenea Inc. (2021). Graphene Oxide Product Datasheet, Graphenea Inc. https://www.graphenea.com.

[31] Djenaoucine L., Picazo A., de la Rubia M.A., Gálvez J.C., Moragues A. (2024). Effect of graphene oxide on the hydration process and macro-mechanical properties of cement. Bol. Soc. Esp. Ceram. Vidr..

[32] Djenaoucine L. (2024). Universidad Politécnica de Madrid Performance Evaluation of Graphene Oxide-Enhanced Cement Mortar and Concrete: Implications for Mechanical Strength and Durability. Ph.D. Thesis.

[33] (2020). Standard Test Method for Compositional Analysis by Thermogravimetry.

[34] Hu C., Han Y., Gao Y., Zhang Y., Li Z. (2014). Property investigation of calcium-silicate-hydrate (C–S–H) gel in cementitious composites. Mater. Charact..

[35] Pane I., Hansen W. (2005). Investigation of blended cement hydration by isothermal calorimetry and thermal analysis. Cem. Concr. Res..

[36] Bhatty J.I. (1986). Hydration versus strength in a portland cement developed from domestic mineral wastes—A comparative study. Thermochim. Acta.

[37] Yang Z., Sun Y., Ma F. (2020). Interlayer spacing of multilayer graphene oxide: Influences of oxygen-containing group density, thickness, temperature and strain. Appl. Surf. Sci..

[38] Lian B., de Luca S., You Y., Alwarappan S., Yoshimura M., Sahajwalla V., Smith S.C., Leslie G., Joshi R.K. (2018). Extraordinary water adsorption characteristics of graphene oxide. Chem. Sci..

[39] Ludwig V., de Mendonça J.P.A., de Lima A.H., da Costa Ludwig Z.M., Junqueira G.M.A., Quirino W.G., Sato F. (2020). Graphene oxide in water: A systematic computational experimental study. Graphene Technol..

[40] Wang Q., Li S., Pan S., Cui X., Corr D.J., Shah S.P. (2019). Effect of graphene oxide on the hydration and microstructure of fly ash-cement system. Constr. Build. Mater..

[41] Li X., Liu Y.M., Li W.G., Li C.Y., Sanjayan J.G., Duan W.H., Li Z. (2017). Effects of graphene oxide agglomerates on workability, hydration, microstructure and compressive strength of cement paste. Constr. Build. Mater..

[42] Zhang X., Zhou S., Zhou H., Li D. (2022). The effect of the modification of graphene oxide with γ-aminopropyltriethoxysilane (KH550) on the properties and hydration of cement. Constr. Build. Mater..

[43] Parveen S., Rana S., Fangueiro R., Paiva M.C. (2015). Microstructure and mechanical properties of carbon nanotube reinforced cementitious composites developed using a novel dispersion technique. Cem. Concr. Res..

[44] Zheng S., Tu Q., Urban J.J., Li S., Mi B. (2017). Swelling of Graphene Oxide Membranes in Aqueous Solution: Characterization of Interlayer Spacing and Insight into Water Transport Mechanisms. ACS Nano.

[45] Holzwarth U., Gibson N. (2011). The Scherrer equation versus the “Debye-Scherrer equation”. Nat. Nanotechnol..

[46] Monteagudo S.M. (2014). Estudio Microestructural y de los Procesos de Hidratación de Cementos con Adiciones. Doctoral Thesis.

[47] Qureshi T.S., Panesar D.K. (2019). Impact of graphene oxide and highly reduced graphene oxide on cement based composites. Constr. Build. Mater..

[48] Li X., Korayem A.H., Li C., Liu Y., He H., Sanjayan J.G., Duan W.H. (2016). Incorporation of graphene oxide and silica fume into cement paste: A study of dispersion and compressive strength. Constr. Build. Mater..

[49] Li W., Li X., Chen S.J., Liu Y.M., Duan W.H., Shah S.P. (2017). Effects of graphene oxide on early-age hydration and electrical resistivity of Portland cement paste. Constr. Build. Mater..

